# Virus-induced ER stress and the unfolded protein response

**DOI:** 10.3389/fpls.2012.00293

**Published:** 2012-12-28

**Authors:** Lingrui Zhang, Aiming Wang

**Affiliations:** Southern Crop Protection and Food Research Centre, Agriculture and Agri-Food CanadaLondon, ON, Canada

**Keywords:** virus, endoplasmic reticulum, ER stress, unfolded protein response, signaling transduction

## Abstract

The accumulation of unfolded or misfolded proteins in the lumen of the endoplasmic reticulum (ER) results in ER stress that triggers cytoprotective signaling pathways, termed the unfolded protein response (UPR), to restore and maintain homeostasis in the ER or to induce apoptosis if ER stress remains unmitigated. The UPR signaling network encompasses three core elements, i.e., PKR-like ER kinase (PERK), activating transcription factor 6 (ATF6), and inositol-requiring protein-1 (IRE1). Activation of these three branch pathways of the UPR leads to the translation arrest and degradation of misfolded proteins, the expression of ER molecular chaperones, and the expansion of the ER membrane to decrease the load of proteins and increase the protein-folding capacity in the ER. Recently, the essential roles of the UPR have been implicated in a number of mammalian diseases, particularly viral diseases. In virus-infected cells, the cellular translation machinery is hijacked by the infecting virus to produce large amounts of viral proteins, which inevitably perturbs ER homeostasis and causes ER stress. This review summarizes current knowledge about the UPR signaling pathways, highlights two identified UPR pathways in plants, and discuss progress in elucidating the UPR in virus-infected cells and its functional roles in viral infection.

## Introduction

The endoplasmic reticulum (ER) is a membrane-bound compartment that plays important roles in many cellular processes such as calcium homeostasis and protein processing (Kim et al., 2008; Hetz et al., 2011; Hetz, 2012). Secretory and membrane proteins are synthesized on ribosomes and translocated in an unfolded state into the ER lumen, where they undergo folding, organelle-specific post-translational modifications, and assembly into higher-order structures (Ellgaard and Helenius, 2003; He and Klionsky, 2009; Marcinak and Ron, 2010). As an organelle for folding and modifications of proteins, the ER is loaded with extremely high concentration of proteins (>100 mg/ml), a concentration at which co-aggregation between proteins and/or polypeptides is clearly promoted (Stevens and Argon, 1999). Therefore, the lumen of the ER needs a unique cellular environment that promotes processing and prevents aggregation (Anelli and Sitia, 2008; Kim et al., 2008; Hetz et al., 2011; Hetz, 2012). Indeed, as the major intracellular calcium pool, the ER is the proximal site of a signal transduction cascade that serves to keep cellular homeostasis (Hendershot, 2004; Kim et al., 2008; Hetz et al., 2011; Hetz, 2012). It is also rich in calcium-dependent ***molecular chaperones*** (see “Glossary”) such as ER luminal binding proteins (BiP), calmodulin (CAM), and calreticulin (CRT), which assist in *de novo* folding or refolding of proteins with high fidelity (Navazio et al., 2001; Ellgaard and Helenius, 2003; Seo et al., 2008). Furthermore, the ER lumen has an oxidative environment, which is essential for ***protein disulphide isomerase*** (PDI)-mediated disulfide formation (see “Glossary”), a process required for the proper folding of a variety of proteins (Kim et al., 2008).

**Figure 1:**
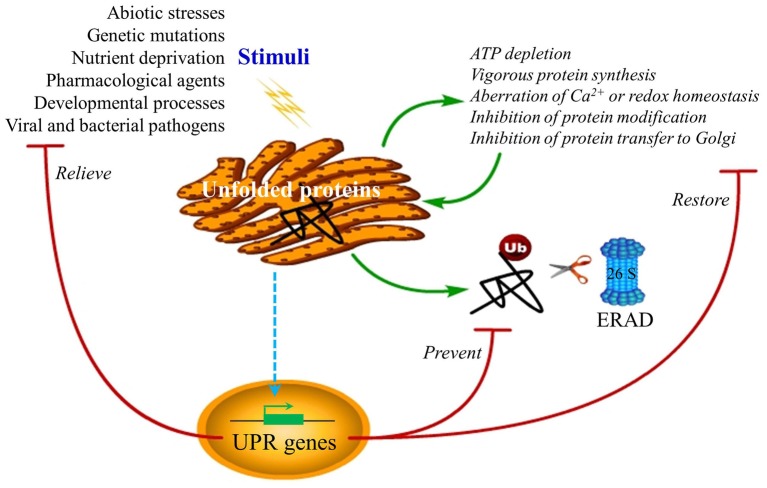
**ER stress and UPR functions.** Disturbances of ER homeostasis cause overload of unfolded or misfolded protein in the ER lumen, a condition termed ER stress, triggering the UPR. The UPR may be induced by pharmacological chemicals, such as tunicamycin, thapsigargin, homocysteine, reductive/oxidative agents as well as non-steroidal anti-inflammatory agents, which impose stress on the ER by causing the vigorous protein synthesis, the imbalance of ER Ca^2+^ and redox, and the inhibition of protein modification or transfer to the Golgi body. In mammalian cells, ER stress also occurs under many circumstances, such as nutrient deprivation, developmental processes, genetic mutation, as well as pathogenic insult. The best-known example of ER stress arising from genetic mutation is the protein-misfolding diseases in human. Recent reports in plants have indicated a close connection between the UPR and environmental stimuli such as heat, salt, and drought stress as well as viral attack, although the underlying mechanisms are largely unknown. The purpose of the induced UPR is to restore the ER function and relive the stress exerted on the ER. In addition, the UPR also eliminates the cytotoxic malformed proteins, which are dislocated across the ER membrane for ubiquitination (Ub) and proteasome-mediated degradation through a pathway known as ERAD. However, if ER homeostasis or function cannot be re-established, programmed cell death will be activated by the UPR, presumably to protect the organism from the rogue cells that display misfolded proteins, which has not yet been confirmed in plants and is not shown in the diagram.

However, the load of client proteins may exceed the assigned processing capacity of the ER due to physiological fluctuations in the demand for protein synthesis and secretion (Zhang and Kaufman, 2006; Ron and Walter, 2007; Marcinak and Ron, 2010; Hetz et al., 2011). The resulting imbalance is referred to as ***ER stress*** (Figure 1) (see “Glossary”), which is a pervasive feature of eukaryotic cells (Gao et al., 2008; Liu and Howell, 2010; Marcinak and Ron, 2010; Hetz et al., 2011; Iwata and Koizumi, 2012). In yeast, animals, and plants, ER stress arises under various circumstances (Figure 1), including developmental processes that affect protein homeostasis networks and genetic mutations that erode the functionality of the ER (Brewer and Hendershot, 2004; Schröder and Kaufman, 2005; Balch et al., 2008; Kim et al., 2008; Marcinak and Ron, 2010; Hetz et al., 2011). In fact, a variety of external stimuli (abiotic and biotic stress) such as pathogen invasion, chemical insult, and energy or nutrient (glucose) deprivation have been shown to impose stress on the ER by leading to alterations of cellular redox equilibrium, disturbances of calcium homeostasis, failure of post-translational modifications, and a general increase in protein synthesis (Figure 1) (Dimcheff et al., 2004; Ye et al., 2011; Iwata and Koizumi, 2012). In general, perturbation of ER homeostasis causes unfolded proteins to accumulate in the lumen of the ER, triggering an evolutionarily conserved cytoprotective signaling pathway designated as the ***unfolded protein response*** (UPR) (Figure 1) (see “Glossary”) (Zhang and Kaufman, 2006; Ron and Walter, 2007; Urade, 2007; Kim et al., 2008).

The initial intent of the UPR is to reestablish homeostasis, relieve stress exerted on the ER, and prevent the cytotoxic impact of malformed proteins via inhibition of mRNA translation and activation of adaptive mechanisms (Figure 1) (Xu, 2005; Kim et al., 2008; Preston et al., 2009; Ye et al., 2011). The adaptation effect predominantly refers to the upregulation of particular groups of genes to enhance the protein folding capacity of the ER and to promote ***ER-assisted degradation*** (ERAD) (see “Glossary”) (Meusser et al., 2005; Kim et al., 2008). The signal-transduction events that are commonly associated with innate immunity and host defense, including mitogen-activated protein kinases (MAPKs), c-Jun N-terminal kinase (JNK), p38, and other kinases responsible for activation of nuclear factor-κB (NF-κB), are also induced, known as UPR-induced alarm mechanisms (Kaneko et al., 2003; Xu, 2005; Kim et al., 2008; Tabas and Ron, 2011). However, if the function of the ER cannot be reestablished especially under the conditions when the primary stimuli causing protein unfolding are excessive and/or protracted, a final mechanism called ***programmed cell death*** (also apoptosis in animals) (see “Glossary”) is triggered, which presumably helps protect the organism from the expansion of potentially harmful substances produced by the damaged cells (Zhao and Ackerman, 2006; Ron and Walter, 2007; Kim et al., 2008; Tabas and Ron, 2011). The ER stress-induced cell death pathway is conserved throughout the plant and animal kingdoms (Urade, 2007; Qiang et al., 2012; Ye et al., 2012). In *Arabidopsis thaliana* roots, the mutualistic fungus *Piriformospora indica* induces ER stress but inhibits the adaptive UPR, resulting in a caspase 1-like mediated cell death, which is required for the establishment of the symbiosis (Qiang et al., 2012).

There is not only an increasing biomedical interest in but also a strong practical demand for investigating the molecular mechanisms underlying the UPR and the development of strategies to manipulate this pathway, due to the fact that chronic ER stress is involved in a number of mammalian diseases including cancers, neurodegeneration, diabetes, inflammation, atherosclerosis, and renal and viral diseases (He, 2006; Zhao and Ackerman, 2006; Yoshida, 2007; Hetz et al., 2011; Tabas and Ron, 2011). The molecular mechanism of the UPR has been investigated extensively in yeast and animals and to a much lesser extent in plants (Cox and Walter, 1996; Sidrauski and Walter, 1997; Oikawa et al., 2010). In mammalian cells, the UPR is mediated by two types of ER transmembrane proteins (ER stress sensors). The type I ER stress sensor consists of IRE1 (inositol-requiring transmembrane kinase/endonuclease) including two identifiable IRE1 isoforms IRE1α and IRE1β, and PERK (PKR-like ER kinase), whereas the type II ER stress sensor includes ATF6α and ATF6β (activating transcription factor 6) (Hetz et al., 2011). In contrast to animals, the UPR in yeast is controlled by only one signaling pathway, the type I transmembrane ER protein IRE1p (Cox and Walter, 1996; Sidrauski and Walter, 1997; Oikawa et al., 2010).

In the past several years, the plant UPR signaling pathway has begun to be explored (Urade, 2007; Vitale and Boston, 2008; Deng et al., 2011; Nagashima et al., 2011). Thus far, two UPR pathways have been identified in plants, one mediated by IRE1-bZIP60 (basic leucine zipper), and the other by bZIP17/bZIP28 which is analogous to the animal ATF6 pathway (Urade, 2007; Vitale and Boston, 2008; Deng et al., 2011; Nagashima et al., 2011). In addition, an adaptive pathway mediated by plant-specific N-rich proteins, which diverges from the molecular chaperone-inducing branch of the UPR, was described as a novel branch of the ER stress response in plants that shares components with the osmotic stress signaling (Costa et al., 2008). Much of the work in plants has concentrated on ER stress induced by environmental cues (Iwata and Koizumi, 2012). For instance, in response to heat stress, two UPR pathways were found to be activated, indicated by bZIP28 proteolytic activation and bZIP60 mRNA splicing (Gao et al., 2008; Deng et al., 2011). The UPR and salt or drought stress have drawn attention from several laboratories (Irsigler et al., 2007; Liu et al., 2007; Costa et al., 2008; Liu and Howell, 2010). More recently, the essential role of the UPR in plants in response to viral attack has also been investigated (Ye and Verchot, 2011; Ye et al., 2011, 2012). In this review, we summarize in detail the current proposed models of how the ER transmembrane proteins sense the unfolded settings, and then address primarily the mechanistically distinct arms of the UPR as well as their relevance to viral infection in animals and plants. Some UPR related proteins such as cellular chaperons and folding enzymes may directly participate in the formation of membrane bound replication and movement complexes. Interested readers may refer to another review published in this special issue (Verchot, 2012). Finally, we discuss possible future directions of research on plant UPR, especially its roles in viral infection.

## BiP: the suppressor of the UPR?

It is generally accepted that signaling in the UPR is initiated by UPR stress sensors, which are ER resident transmembrane proteins. They utilize their luminal portions to sense the protein-folding environment in the ER, and their cytoplasmic effector portions to interact with the transcriptional or translational apparatus (Ron and Walter, 2007). To date, several models have been proposed to explain how the unfolded protein load is detected by ER stress transducers (UPR stress sensors) to initiate the UPR activation (Parmar and Schröder, 2012).

### Indirect recognition model

**Figure 2:**
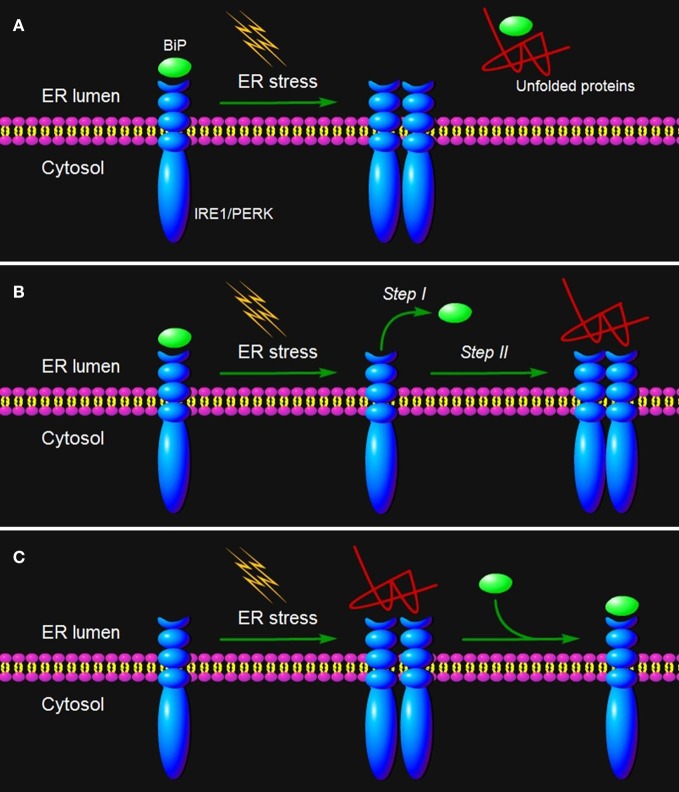
**ER stress sensing mechanism by IRE1/PERK.** Three models are proposed to explain IRE1/PERK activation in response to the accumulation of unfolded proteins in the ER lumen. **(A)** The indirect recognition model proposes that BiP binding maintains IRE1/PERK in an inactive monomeric state. During ER stress, BiP is dissociated from its partners to bind unfolded proteins, which leads to the spontaneous dimerization of IRE1/PERK and activation of their RNase domains. In this case, BiP operates as the “UPR master control/ER stress sensor.” The model may also operate in the control of ATF6 activation. **(B)** The semi-direct recognition model summarizes findings from studies of IRE1p in yeast and analyses of IRE1 crystal structure. This model proposes that the IRE1 is activated via two steps. In the first step, BiP dissociation from IRE1 leads to formation of higher order oligomers (called cluster). In the second step, direct interaction of unfolded proteins with IRE1 stabilizes the cytosolic domains of clustered IRE1 molecules and thus causes IRE1 activation. **(C)** A direct recognition model outlines recent studies in yeast. Three subpopulations of IRE1p co-exist within the cell: an inactive pool in equilibrium with an active unfolded protein-bound pool. The latter is sequestered by BiP binding, designated the third inactive set. In this model, BiP binding to or release from IRE1p does not activate the UPR, but it may serve as a buffer and a timer to adjust the sensitivity and dynamics of IRE1p activity. In turn, the unfolded protein binding to IRE1 is the single step of its activation.

The ER chaperone immunoglobulin heavy-chain BiP, also known as glucose-regulated protein 78 (GRP78), has been proposed as a master repressor of UPR (Hendershot, 2004; He, 2006; Zhang and Kaufman, 2006; Parmar and Schröder, 2012). It has been long known that BiP is more strongly induced by slowly folding proteins with a prolonged interaction with BiP than fast folding proteins (Gething et al., 1986; Watowich et al., 1991; Kohno et al., 1993). In normal cells, BiP keep UPR stress sensors in their inactive monomeric states through binding to their luminal domains (Figure 2A). Conversely, in cells undergoing ER stress, BiP is released when sequestered by unfolded proteins, leading to the activation of these ER stress sensors (Figure 2A) (Parmar and Schröder, 2012). Pivotal evidence for this chaperon-mediated model (indirect recognition model) comes from immunoprecipitation assay directly showing that, in unstressed acinar and fibroblasts cells, the luminal domains of PERK and IRE1 form a stable complex with the ER chaperone BiP, and the perturbation of protein folding promotes reversible dissociation of BiP from these two type-I transmembrane protein kinases, which correlates with the formation of activated PERK or IRE1 (Bertolotti et al., 2000). Consistently, in CHO cells stably overexpressing BiP, the amount of BiP being associated with PERK or IRE1 is considerably greater than that in parental CHO cells with normal levels of endogenous BiP (Bertolotti et al., 2000). Moreover, in BiP-overexpressing CHO cells, phosphorylation of PERK is delayed and incomplete, and activation of IRE1α by ER stress is absent (Dorner et al., 1992; Wang et al., 1996; Bertolotti et al., 2000). In fact, the UPR is attenuated by overexpression of only BiP rather than of other UPR molecular signatures (Dorner et al., 1990, 1992). As for the type-II transmembrane transducer, overexpression of wild-type BiP dramatically delays the translocation of ATF6 to the Golgi and leads to the lower amount of cleaved ATF6 in dithiothreitol (DTT)-treated Hela cell (Shen et al., 2002). A BiP mutant that bears a point mutation in its ATPase domain and loose ability to dissociate from ATF6 completely abolishes DTT-induced ATF6 activation (Shen et al., 2002). Collectively, these data suggest that the mechanisms of ER stress sensing by type-I transmembrane sensors may also operate in the control of type-II transmembrane sensor activation.

### Semi-direct recognition model

However, the indirect recognition model is challenged by an observation in yeast that deletion of the BiP-binding site renders IRE1p unaltered in ER stress inducibility, although it abolishes BiP binding (Kimata et al., 2004). The crystal structure of the yeast IRE1p luminal domain suggests that an IRE1p dimer creates a shared central groove formed by α-helices, with an architectural resemblance to the peptide binding domains of ***major histocompatibility complexes*** (MHCs) (see “Glossary”) (Credle et al., 2005; Parmar and Schröder, 2012). Thus, IRE1 itself has the intrinsic ability to sense ER stress, and its activation may be initiated by BiP dissociation and further triggered by binding of unfolded proteins to its luminal domains (Figure 2B) (Kimata et al., 2004). This two-step activation model (semi-direct recognition model, Figure 2B) is proposed considering findings that BiP mutants locked in the ATP-bound state, but not the ADP-bound state interact with IRE1 (Kimata et al., 2003). Analysis of mutation in BiP ATPase domain further revealed that the conformational change in BiP induced by the binding of unfolded proteins to ATP-bound BiP leads to ATP hydrolysis, conversion of BiP to the ADP-bound state and release from IRE1 (Kimata et al., 2003; Todd-Corlett et al., 2007). This model is also supported by the fact that recombinant luminal domains of the yeast IRE1p is associated with unfolded proteins in a cell-free system (Kimata et al., 2007). However, this model remains controversial as there lacks evidence that unfolded proteins bind to IRE1 *in vivo*, and there is no time-course analysis of BiP dissociation and binding of unfolded proteins to IRE1.

### Direct recognition model

Recently, based on time-resolved analysis of IRE1p signaling in yeast, Peter Walter's group has proposed a new quantitative model (direct recognition model, Figure 2C). In this dynamic UPR regulation model, IRE1 is in a dynamic equilibrium with BiP and unfolded proteins, and the unfolded protein binding to IRE1 is the single and sufficient step for activation of the UPR (Pincus et al., 2010). BiP binding to or release from IRE1 is ruled out as the primary switch that governs the UPR on or off as previously proposed, and it might act as a buffer and a timer to fine-tune the sensitivity and dynamics of the UPR, respectively (Figure 2C) (Pincus et al., 2010). The direct recognition model is strengthened by elegant biochemical assays showing that unfolded proteins are IRE1p-activating ligands that could directly induce the UPR in yeast cells (Gardner and Walter, 2011). Binding of unfolded proteins to IRE1 monomers induces dimerization via formation of the MHC-like peptide binding groove (Credle et al., 2005; Gardner and Walter, 2011). Moreover, considerable data suggest that the cluster formation is a prerequisite for signaling by IRE1 (Credle et al., 2005; Kimata et al., 2007; Aragón et al., 2008; Korennykh et al., 2008). Nevertheless, the recombinant luminal regions of human IRE1 do not interact with unfolded proteins in a cell-free system (Oikawa et al., 2009), consistent with a previous prediction that, unlike yeast IRE1p, the MHC-like groove in the crystal structure of human IRE1 is too narrow for peptide binding (Zhou et al., 2006).

The difference in IRE1 structure between yeast and human reminds us that the complexity of ER stress sensing is far beyond our understanding and that structure-functional analysis in this field is far from complete. In the case of plants, the *Arabidopsis* and rice IRE1 proteins are the ER-resident proteins that possess kinase activity and have ability to sense ER stress with their luminal domain (Iwata and Koizumi, 2012). Although it has been known that overexpression of BiP in tobacco and soybean prevents activation of the UPR by ER stress inducers (Leborgne-Castel et al., 1999; Costa et al., 2008), the underlying mechanisms of ER stress sensing by plant IRE1 have not been investigated.

### Viral infection and ER sensing

In the recent decades, the importance of ER stress and UPR response in viral infection has been demonstrated in mammalian cells (Jordan et al., 2002; Baltzis et al., 2004; Netherton et al., 2004; Sun et al., 2004; Tardif et al., 2005). In a productive viral infection, large amounts of viral proteins are synthesized in infected cells, which lead to an overwhelming load of unfolded or misfolded proteins (Kim et al., 2008). Many mammalian viruses have evolved to manipulate host UPR signaling pathways to promote viral translation and persistence in infected cells. For example, flaviviruses such as Japanese encephalitis virus (JEV) and dengue viruses (DEN) trigger the specific UPR pathway, leading to enhanced protein folding abilities (Urano et al., 2000). Early studies with ***hemagglutinin-neuroamindase*** (HN) (see “Glossary”) glycoproteins of influenza virus revealed that BiP associates transiently and non-covalently with the unfolded or immature glycoproteins (Hurtley et al., 1989). The misfolded, BiP-associated glycoproteins are not transported to the plasma membrane but persist as complexes in the ER for a long period of time before degradation (Hurtley et al., 1989). Similar observations have been reported with glycoprotein G of vesicular stomatitis virus, HN glycoproteins of paramyxovirus SV5, and glycoprotein of hepatitis C virus (HCV) (Ng et al., 1989; Machamer et al., 1990; Choukhi et al., 1998). Taken together, these data support the model in which interaction of BiP with unfolded viral proteins triggers the UPR response during viral infection.

Intriguingly, among 7 proteins encoded by simian virus 5, only the HN glycoprotein stimulates UPR response (Hurtley et al., 1989; Watowich et al., 1991). In virus-infected cells, the HN glycoprotein is inserted into the ER, and then transported to cell surface (He, 2006). Similarly, ectopic expression of the E2 protein, but not E1, core and NS3 proteins of HCV activates the expression of BiP (Liberman et al., 1999). HCV replicons expressing only non-structural proteins are also capable of stimulating BiP expression (Tardif et al., 2002). Infection of cytomegalovirus (CMV) causes a transient increase in BiP levels at the early phase of viral replication. Moreover, the expression of CMV *Us11* that physically interacts with BiP in mammalian cells is sufficient to trigger the UPR (Tirosh et al., 2005). In addition, several other studies have also suggested a connection between the UPR and viral replication. These include herpes simplex virus (HSV) 1, JEV, and HCV (Su et al., 2002; Cheng et al., 2005; Tardif et al., 2005). These studies suggest that either the process of viral replication or the production of a specific viral protein in the ER is capable of inducing UPR response.

Although how ER stress sensors sense viral infection to activate the UPR is not clear, a recent study with severe acute respiratory syndrome (SARS) coronavirus (SARS-CoV) has identified one of accessory proteins of SARS-CoV, the 8ab protein that could bind directly to the luminal domain of ATF6, the type II ER stress sensor (Sung et al., 2009). Ectopic expression of the 8ab protein in mammalian cells induces the proteolysis of ATF6 and the translocation of its cleaved DNA-binding and transcription-activation domains from the ER to nucleus (Sung et al., 2009). These findings suggest that viruses may exploit their own protein(s) to directly modulate UPR response.

As has been reported for animals, the most prominent phenomenon in plants induced by the UPR is the transcriptional induction of ER chaperone and protein-folding genes, such as BiP, CRT, and PDI (Schott et al., 2010). Recently, *Arabidopsis* stromal-derived factor 2 (SDF2) was identified as a crucial target of the plant UPR with a direct function in ER protein quality control (Schott et al., 2010). Using a combination of biochemical and cell biological methods, SDF2 was shown to respond to ER stress conditions and pathogen infestation in a manner similar to known molecular UPR markers (Wang et al., 2005; Schott et al., 2010). In plants, microarray-based analyses of gene expression have shown that BiP is upregulated in *Arabidopsis* in response to infections by *Turnip mosaic virus* (TuMV) and *Oilseed rape mosaic virus* (ORMV) (Whitham et al., 2003; Yang et al., 2007; García-Marcos et al., 2009). Similar upregulation of ER-resident chaperones has also been found in *Arabidopsis* and potato (*Solanum tuberosum*) during *Potato virus X* (PVX) infection (Whitham et al., 2003; Yang et al., 2007; García-Marcos et al., 2009). In PVX infection, a viral movement protein TGBp3, which resides in the ER, elicits the UPR in *Arabidopsis* and *Nicotiana benthamiana* as an early response to virus infection (Ye and Verchot, 2011; Ye et al., 2011). Similar to the ER-resident proteins encoded by flaviviruses or retroviruses such as HIV (Tardif et al., 2004; Chan and Egan, 2005; Sung et al., 2009), TGBp3 modulates the UPR signaling as a means to cope with robust viral protein synthesis (Ye and Verchot, 2011; Ye et al., 2011). In the case of HIV, the Vpu protein coded by HIV has been shown to trigger the degradation of the host CD4 protein by the 26S proteasome, and this degradation is vital for virion release (Schubert et al., 1998; Meusser et al., 2005; Nomaguchi et al., 2008). Considering the similarity of TGBp3 to Vpu in terms of molecular mass and subcellular localization, TGBp3 may have analogous functions to Vpu in targeting host proteins for ubiquitination and degradation to ensure virus spread (Ye et al., 2012). In addition, the TGBp3-elicited UPR effectively delays the host immune responses to aid PVX infection, including TGBp3-triggered programmed cell death (Ye et al., 2012). The induction of cell death pathway can be suppressed by overexpression of BiP and is dependent on SKP1, a core subunit of the SCF (SKP1/Cullin1/F-box protein) ubiquitin E3 ligase complex (Ye et al., 2012). However, the mechanisms of the activation of the UPR by TGBp3 in PVX infection or by other viral proteins (if any) in infections by other plant viruses as well as the roles of the chaperone BiP in governing the UPR in virus-infected plants still remain unknown.

## Three pathways of the UPR

### PERK pathway and protein synthesis control

**Figure 3:**
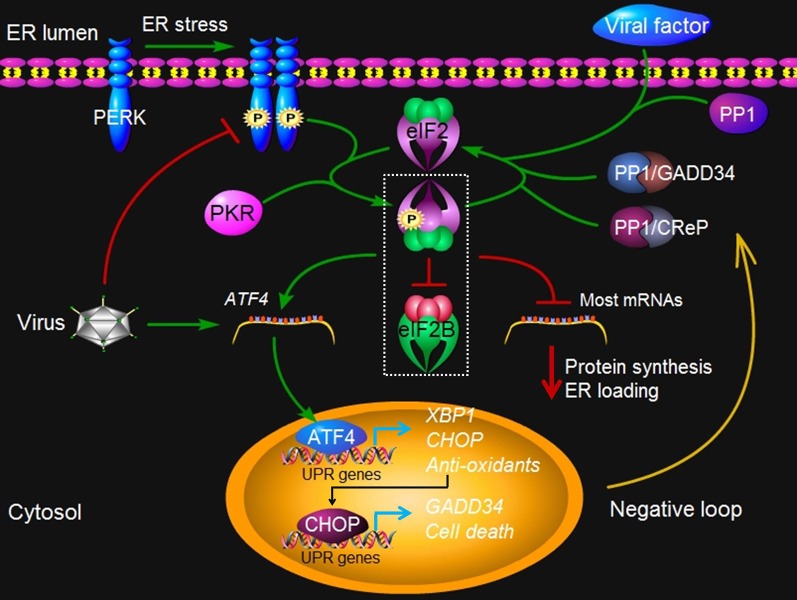
**PERK signaling under virus attack.** Upon ER stress such as virus infection, protein kinase PERK oligomerizes in the ER membrane and is activated via *trans*-autophosphorylation. The activated PERK phosphorylates a subunit of eIF2, which inhibits the exchange factor eIF2B from recycling eIF2 to its active GTP-bound form. In addition, dsRNA-activated protein kinase R (PKR) can also activate this pathway independently of PERK. The resulting reduced activities of eIF2B and the eIF2 complex account for all of the important consequences of PERK activity, such as translation inhibition of most mRNAs, which reduces protein synthesis and lowers ER loading. However, some mRNA such as *ATF4* gains a selective advantage for translation via phosphorylated eIF2. ATF4 in turn contributes to the transcriptional activation of *CHOP*, *XBP1*, *GADD34*, and other genes involved oxidative stress and cell death. GADD34 is a regulatory subunit of protein phosphatase (PP) 1 that dephosphorylates eIF2α and recovers the activity of eIF2, constituting a negative feedback loop for regulation of PERK signaling. A constitutive phosphatase CreP also promotes eIF2 dephosphorylation. Viruses such as CMV may directly exploit the negative loop to terminate the PERK signaling pathway, via increasing the expression of ATF4, because the prolonged closure of protein synthesis is harmful to virus infection. Some viruses, such as HSV1 and ASFV, may produce a viral factor, which is homologous to host GADD34, to restore the activity of eIF2 along with PP1. Other viruses such as HCV may encode a viral protein that binds to PERK as a pseudosubstrate and thus, inhibits PERK activation. Finally, viruses such as LCMV may selectively activate the branches of the UPR to favor their replication. At present, no PERK-like pathway has been found in plants.

PERK is a ER-localized type I transmembrane protein, with a catalytic kinase domain sharing substantial homology to other kinases of the ***eukaryotic translation initiation factor 2*** (eIF2) (see “Glossary”) (Harding et al., 1999). In the early phase of ER stress, accumulation of unfolded or misfolded protein leads to oligomerization of PERK in the ER membranes, inducing its *trans*-autophosphorylation and kinase domain activation (He, 2006) ER stress-activated PERK phosphorylates eIF2α on Ser51, which inhibits the guanine nucleotide exchange factor eIF2B from recycling eIF2 to its active GTP-bound form (Figure 3). As a result, mRNA translation is shut off and the load of newly synthesized proteins is reduced that are destined to enter the already stressed ER lumen (Figure 3) (Hetz et al., 2006). An exceptional case to this general response is that certain mRNAs gain a selective advantage for translation under conditions in which eIF2α is phosphorylated (Figure 3) (Lu et al., 2004). The 5′ untranslated region of these mRNA contains short, inhibitory upstream open reading frames (uORFs) that prevent translation of their downstream encoding ORF in unstressed cells. When eIF2α activity is limited due to its phosphorylation in stressed cells, ribosomes skip the inhibitory uORFs so that they can be translated (Ron and Walter, 2007). Two of such genes that have been extensively studied include the transcription factor Gcn4 (general control non-depressible-4) in yeast and ATF4 in mammalian cells (Figure 3) (Hinnebusch and Natarajan, 2002; Lu et al., 2004; Vattem and Wek, 2004). ATF4 is responsible for stimulating the expression of a pro-apoptotic factor C/EBP homologous protein (CHOP), as well as growth arrest and DNA damage-inducible protein 34 (GADD34) (Figure 3) (Zinszner et al., 1998; Novoa et al., 2003).

A chemical inhibitor that sustains phosphorylation of eIF2α protects rat pheochromocytoma cell from ER stress, suggesting that the maintenance of eIF2α in an inactive state is somehow beneficial to cell survival during the circumstances that induce ER stress (Boyce et al., 2005). However, prolonged suppression of protein synthesis is typically incompatible with cell survival (Ron and Walter, 2007; Kim et al., 2008). Although the regulatory mechanisms and the phosphatase(s) involved are yet to be characterized, it has been reported that ER stress-induced PERK activation in pancreatic AR42J cells is rapidly reversible, and, upon removal of ER stress, activated PERK is dephosphorylated (Bertolotti et al., 2000; Jousse et al., 2003). In fact, it is well known that phosphorylated eIF2α is also subject to negative regulation (Ron and Walter, 2007). Somatic-cell genetic screen has identified two genes GADD34 and CReP (constitutive repressors of eIF2α phosphorylation) encoding the substrate targeting subunits of two phosphatase complexes that independently dephosphorylate eIF2α (Figure 3) (Connor et al., 2001; Jousse et al., 2003; Ma and Hendershot, 2003). CReP is constitutively expressed and contributes to baseline eIF2α dephosphorylation, whereas GADD34 is induced as part of the gene expression program activated by eIF2α phosphorylation and serves in a negative feedback loop that regulates eIF2α activity (Figure 3) (Jousse et al., 2003; Novoa et al., 2003).

In mammalian cells, a considerable body of evidence has indicated the association of viral replication with the PERK pathway (Jordan et al., 2002; Baltzis et al., 2004; Netherton et al., 2004; Sun et al., 2004; Boyce et al., 2005; Cheng et al., 2005; Isler et al., 2005). It becomes clear that the battle between the invading virus and the host cell in the ER is complicated. The repair of the ER function offered by PERK activation is beneficial to viral replication (He, 2006). On the other hand, the inhibition of protein synthesis mediated by the PERK pathway conversely regulates viral replication and maturation as all viruses depend on the cell translation machinery to synthesize viral proteins. Then one may wonder how viruses manage to overcome the translation inhibition imposed by the PERK pathway for the high speed production of viral proteins required for virus multiplication.

In human and mouse cells infected with the DNA virus HSV1, the production and processing of viral proteins in the ER presumably trigger the oligomerization of PERK, leading to the activation of PERK, as estimated by an increase in autophosphorylation of PERK (Cheng et al., 2005). Interestingly, in these cells with activated PERK, eIF2α remains in the unphosphorylated state, and viral polypeptide synthesis is thus normal. Obviously, the virus stimulates and then disarms the PERK activity. A virulence factor, the γ134.5 protein encoded by HSV1, has been shown to have a critical role in mediating eIF2α dephosphorylation in virus-infected cells (Figure 3) (He et al., 1997; Cheng et al., 2005). Furthermore, the γ134.5 protein can alleviate the translation arrest caused by the UPR inducing compounds DTT and thapsigargin (He et al., 1997; Cheng et al., 2005). Importantly, the γ134.5 protein also inhibits the activity of double-stranded RNA-dependent protein kinase R (PKR) by mediating eIF2α dephosphorylation (Figure 3) (He et al., 1997, 1998; Cheng et al., 2001). Indeed, the carboxyl-terminal domain of viral γ134.5 protein is highly homologous to the corresponding region of GADD34, suggesting the domain shared by the two proteins may perform a common function (He et al., 1997; Cheng et al., 2005). Like GADD34, the γ134.5 protein can recruit protein phosphatase 1 to dephosphorylate eIF2α and block translation shutoff during viral infection (Figure 3) (He et al., 1997; Cheng et al., 2005). Together, these findings suggest that the viral protein γ134.5 functions as an antagonist to the inhibitory activity of the PERK pathway on protein translation by maintaining the eIF2 activity during a productive HSV1 infection.

Although ER stress and the UPR are evident in the course of productive infection by African swine fever virus (ASFV, DNA virus), PERK activation seems not to be induced (Galindo et al., 2012). In Vero (African green monkey kidney) cells infected by ASFV, the eIF2α phosphorylation is maintained at a lower level in order to restore protein translation (Galindo et al., 2012). Furthermore, ASFV is capable of blocking the expression of CHOP induced by DTT, thapsigargin, and other agents (Netherton et al., 2004). ASFV also encodes the viral protein DP71L, a homolog to GADD34 (Zsak et al., 1996). However, it is not clear if DP71L also involves in the inhibition of PERK activation.

It is well documented that the human DNA virus CMV perturbs the PERK pathway (Netherton et al., 2004; Isler et al., 2005; Tirosh et al., 2005). Unlike HSV1, CMV replicates slowly and in an ordered temporal manner. It seems that CMV directly exploits the cellular negative feedback loop to inhibit PERK activities. In human foreskin fibroblasts (HFFs) cells infected with CMV, PERK is not phosphorylated in the early phase. As viral replication proceeds, there is an increase in the level of PERK phosphorylation. However, the amount of phosphorylated eIF2α is limited and translation attenuation does not occur (Netherton et al., 2004; Isler et al., 2005; Tirosh et al., 2005). Interestingly, translation of ATF4, which is dependent on eIF2α phosphorylation, is significantly increased (Netherton et al., 2004; Isler et al., 2005; Tirosh et al., 2005). Expression of ATF4 leads to the activation of target genes involved in the maintenance of metabolism and redox state, and thus may benefit CMV infection by maintaining a permissive cellular environment (Figure 3). It is worth to note that ATF4-induced GADD34 can act directly downstream of eIF2α phosphorylation to eliminate the negative effects of PERK activation (Figure 3) (Jousse et al., 2003; Novoa et al., 2003).

The PERK pathway is also associated with infections by RNA viruses. For example, a cytopathic strain of bovine viral diarrhea virus (BVDV), a member of flaviviruses, activates PERK and causes hyperphosphorylation of eIF2α (Jordan et al., 2002). However, it remains unclear as to how the translation attenuation resulting from PERK activation is overcome by BVDV. HCV encodes a viral E2 protein, which binds to PERK as a pseudosubstrate and may sequester it from its normal substrate eIF2α (Figure 3) (Pavio et al., 2003). Consistently, ectopic expression of the E2 protein inhibits PERK phosphorylation and enhances translation, contributing to a persistent HCV infection. Additionally, viruses such as LCMV (lymphocytic choriomeningitis virus) bypass the PERK pathway to selectively activate the ATF6 pathway (Pasqual et al., 2011). Therefore, different viruses may adapt different strategies to cope with the PERK pathway for a productive infection. To date, no genes homologous to the animal PERK have been found in plants. It is reasonable to speculate that plants do not have the PERK pathway (Iwata and Koizumi, 2012).

### IRE1 pathway and protein degradation

**Figure 4:**
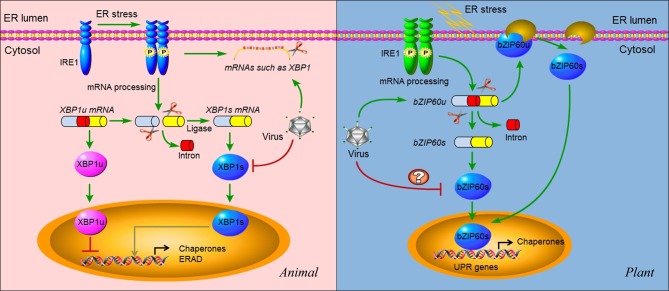
**IRE1 signaling and virus infection in animals and plants.** In animals, IRE1 oligomerizes in the plane of the ER membrane in stressed cells, leading to *trans*-autophosphorylation and activation. Activated IRE1 mediates the sequence-specific cleavage of the XBP1 mRNA in higher eukaryotes, deleting a small RNA fragment (intron) and finally producing a spliced mRNA (*XBP1s*) with a frame shift in the coding sequence. Spliced *XBP1s* encodes a potent transcriptional activator (XBP1s), whereas the unspliced XBP1 mRNA (*XBP1u*) encodes an inhibitor of the UPR (XBP1u). In mammals, it seems that XBP1s regulates a subset of UPR genes that promote ERAD of misfolded proteins and refold proteins. In cultured *Drosophila melanogaster* cells, activated IRE1 can promote the cleavage of mRNAs, including *XBP1* mRNA, leading to their degradation. This reduces the load on the stressed ER and might facilitate reprogramming of the ER-associated protein synthesis and translocation machinery. In cells infected by viruses such as HCV, the IRE1 pathway is manipulated by the virus via repressing the transcriptional activity of XBP1s. In addition, some viruses might also promote the IRE1-dependent mRNA decay as a means to manipulate the IRE1 pathway. In plants, IRE1 homologs were detected in the genomes of *Arabidopsis* and rice a decade ago. However, the target of IRE1 was not identified until 2011. The mRNA of transcriptional factor bZIP60 is the substrate of IRE1 in plants. Similar to *XBP1* in animals, unspliced *bZIP60* (*bZIP60u*) is processed by activated IRE1. The protein product (bZIP60s) translated from the spliced *bZIP60* (*bZIPs*) is translocated into the nucleus to activate the expression of UPR genes such as chaperones. Different from XBP1u, plant bZIP60u protein, translated from *bZIP60u* mRNA, is retained in the ER membrane. Sensing unfolded proteins in the ER lumen, bZIP60u undergoes a proteolytic processing, releasing bZIP60s. A recent study has shown that the expression of bZIP60 was increased by PVX infection. However, the roles of the UPR pathway in virus infection have only begun to be investigated in plants. Critical unanswered questions need to be addressed in the future, such as whether viruses modulate the IRE1 pathway via inhibiting the transcriptional activity of bZIP60s (indicated by “?”).

IRE1, the first UPR transducer identified by a mutation screen in yeast, is a bifunctional enzyme, i.e., a Ser/Thr protein kinase and a site-specific carboxyl-terminal endoribonuclease. Like PERK, IRE1 has an ER luminal amino-terminal domain and a transmembrane domain that anchors IRE1 to the ER membrane (Figure 4) (He, 2006). In response to ER stress, IRE1 is activated directly and/or indirectly by unfolded proteins as mentioned earlier. Unlike PERK, IRE1 signaling does not have selected downstream kinase targets because the only known substrate of the IRE1 kinase is IRE1 itself (Shamu and Walter, 1996; Papa et al., 2003). *Trans*-autophosphorylation of the kinase domain of IRE1 activates its unusual effector function that catalyzes the ***unconventional processing*** (see “Glossary”) of the only known substrate (Figure 4): an mRNA that encodes a UPR transcriptional activator named Hac1 (homologous to ATF/CREB1) in yeast (Cox and Walter, 1996; Mori et al., 1996) or XBP1 (X-box BiP-1) in metazoans (Yoshida et al., 2001; Calfon et al., 2002).

The precursor XBP1 or Hac1 mRNA is cut twice by the activated IRE1, and a 26 nucleotide intron of *xbp1* mRNA is spliced out (Hetz et al., 2011). The 5′ and 3′ mRNA fragments are then re-ligated, producing a spliced mRNA that encodes a 41 kDa XBP1 protein, a bZIP family transcription factor (Figure 4) (Sidrauski et al., 1996; Stephens et al., 2005; Kim et al., 2008). The spliced version of XBP1 (termed XBP1s) upregulates a general population of UPR-related genes mainly involved in protein folding and ERAD (Figure 4) (Lee et al., 2003; Shaffer et al., 2004). Thus, the IRE1-XBP1 pathway directs both protein refolding and degradation in response to ER stress. Recently, the IRE1-dependent degradation of ER-associated mRNAs has also been observed in ER-stressed *Drosophila melanogaster* cells (Hollien and Weissman, 2006; Hollien et al., 2009), allowing to propose an XBP1-independent post-transcriptional mechanism for IRE1 to regulate gene expression that remodels the protein repertoire (Figure 4). However, it is unknown whether the mRNA degradation is promoted by IRE1 with its own endonuclease activity. In fact, in metazoans both the precursor and spliced form of XBP1 are translated (Figure 4) (Calfon et al., 2002; Yoshida et al., 2006). The XBP1s is more stable, working as a transactivator of UPR target genes, whereas the unspliced XBP1 (designated XBP1u) is labile and inhibits transcription of UPR target genes (Figure 4) (Yoshida et al., 2001; Calfon et al., 2002). By contrast, in yeast, the translation of unspliced HAC1 mRNA is repressed due to the presence of intron, and relief of this repression is the key step in activating the yeast UPR (Rüegsegger et al., 2001).

In human hepatoma cells expressing HCV subgenomic replicons, IRE1 is activated as indicated by elevated accumulation and expression of XBP1s (Tardif et al., 2004). However, the *trans*-activating activity of XBP1s is inhibited and the degradation of misfolded proteins is repressed due to the block of ERAD activity. In addition, in an IRE1-null cell line with a defective IRE1-XBP1 pathway, there is an elevated level of translation mediated by the HCV IRES (internal ribosome entry site), which directs the translation of HCV non-structural proteins (Tardif et al., 2004). Based on these data, it is concluded that HCV may suppress the IRE1-XBP1 pathway to stimulate HCV expression and to contribute to the persistence of the virus in infected hepatocytes (Tardif et al., 2004). However, the underlying mechanism of the repression of the transcriptional activity of XBP1s by HVC (Figure 4) is unclear. One possible explanation is that in cells carrying HCV replicons, XBP1 itself is targeted for proteasomal degradation, limiting its transcriptional regulation activity (Trujillo-Alonso et al., 2011). However, how HCV replicons direct XBP1 to be degraded remains to be understood. In addition to post-transcriptional modification by IRE1, HAC1 and XBP1 are also regulated by the UPR as transcriptional targets. In yeast, HAC1 mRNA production is induced by ER stress (Leber et al., 2004). In metazoan cells, levels of XBP1 mRNA also increase upon UPR induction (Yoshida et al., 2006), leading to accumulation of newly transcribed XBP1 mRNAs in their unspliced form. Therefore, the accumulated XBP1u mRNA may serve as an inhibitor to suppress the IRE1 signaling pathway since the XBP1u is a transcriptional repressor of UPR target genes (Yoshida et al., 2001; Calfon et al., 2002). Moreover, the XBP1u mRNA itself may also terminate the IRE1 signaling pathway by inhibitory heterodimerization with spliced XBP1 and/or competition for binding sites (Yoshida et al., 2006), conferring a switch-like property to XBP1-mediated gene regulation. Thus far, however, it is unknown if HCV infection increases the level of XBP1u mRNA and thus suppresses the transcriptional activity of XBP1s. Similar to the case of HCV, infection with human CMV or animal SARS-CoV also leads to a progressive increase in XBP1s mRNA; however, its target genes are not induced, suggesting that either the translation or the transcriptional regulation activity of XBP1s is blocked (Isler et al., 2005; Bechill et al., 2008).

A recent study in lung epithelial cell has showed that influenza A virus activates the IRE1 pathway with little or no concomitant activation of the PERK and ATF6 pathways, and inhibition of IRE1 activity leads to decreased viral replication, suggesting that IRE1 is a potential therapeutic target for influenza A virus (Hassan et al., 2012). In this study, influenza A virus replication also leads to an increase in XBP1 mRNA splicing, which can be blocked by the specific inhibitors of the IRE1 pathway. However, it is unclear if activation of IRE1 but inhibition of XBP1s is also used by influenza A virus as a strategy to cope with the IRE1 activation-mediated antiviral responses. In the case of West Nile Virus (WNV), the IRE1-XBP1 pathway is non-essential for its replication, although XBP1s is induced (Medigeshi et al., 2007). In *xbp1*^−/−^ cells, WNV accumulation is similar to that in the wild type cells, suggesting a possibility that other UPR pathways can compensate for the absence of XBP1 in these cells (Medigeshi et al., 2007). In agreement with these findings, knockdown of XBP1 expression by small interfering RNA has minimal effects on cells' susceptibility to other flaviviruses such as JEV and DEN (Zhao and Ackerman, 2006), although IRE1-XBP1 pathway was activated during the two viruses infection, as evidenced by XBP1 mRNA splicing and protein expression, as well as induction of the downstream genes *ERdj4*, *EDEM1*, and *p58(IPK)* (Yu et al., 2006).

It has been almost one decade since IRE1 homologs were detected in the genomes of *Arabidopsis* and rice (Koizumi et al., 2001; Okushima et al., 2002). Now, it is clear that the mRNAs of *Arabidopsis* bZIP60 (AtbZIP60) and its rice ortholog OsbZIP50, collectively called bZIP60, are spliced by IRE1 (Figure 4) (Deng et al., 2011; Nagashima et al., 2011). The bZIP60 mRNA shares similar secondary structure with HAC1 and XBP1 mRNA, and they also share a similar splicing mechanism (Figure 4) (Iwata and Koizumi, 2012). Besides being processed conventionally as the mRNA targets of IRE1, which seems conserved in both plants and animals, plant bZIP60 has a unique post-translational modification (Iwata and Koizumi, 2005; Iwata et al., 2008). Plant bZIP60 (unspliced) is synthesized at a low level as a precursor protein, which is anchored in the ER membrane under normal conditions (Figure 4). Sensing ER stress by an as yet to be elucidated mechanism, the N-terminal domain of AtbZIP60 is cleaved and translocated to the nucleus (Figure 4) (Iwata and Koizumi, 2005; Iwata et al., 2008, 2009). In turn, the nuclear-localized AtbZIP60 forms a transcriptionally active protein complex of approximately 260 kDa to activate the transcription of UPR genes, such as BiP3, via the *cis*-elements plant-UPR element and ER stress response element (Urade, 2007; Iwata et al., 2009). However, the truncated species of bZIP60 has recently been suggested to be the product translated from the spliced mRNA mediated by IRE1, not the cleaved product of the full-length bZIP60 (Deng et al., 2011; Nagashima et al., 2011). Recently, the role of the bZIP60-mediated UPR has also been demonstrated for the first time in infection by a plant virus. In response to PVX infection or PVX TGBp3 induced-ER stress, bZIP60 is upregulated (Figure 4). Silencing *bZIP60* leads to the suppression of the UPR transcript levels and reduces PVX accumulation (Ye et al., 2011). It is suggested that the bZIP60-mediated UPR may be important to regulate cellular cytotoxicity and beneficial to PVX pathogenesis (Ye et al., 2011). However, the mechanism by which bZIP60 is manipulated by the virus and how bZIP60 operates in induction of the UPR are not clear.

### ATF6 and ER chaperone expression

**Figure 5:**
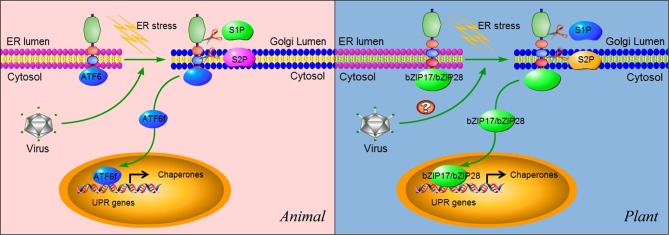
**ATF6 and bZIP17/bZIP28 pathways.** In unstressed cells, ATF6 in animals and bZIP17/bZIP28 in plants reside in the ER membrane. They are delivered to the Golgi apparatus in an unknown mechanism upon sensing ER stress. In the Golgi apparatus, these proteins are subject to cleavage twice, first by the lumenal S1P and then the intra-membrane S2P, to release the cytosolic effector portions of the proteins (ATF6f). ATF6f then enters into the nucleus and probably activates a subset of UPR target genes, although these remain to be characterized. Some viruses such as ASFV have been shown to selectively activate the ATF6 pathway for their replication in animals. In plants, the cleaved N terminal portions of bZIP17 and bZIP28 also move into the nucleus and activate UPR genes. In plants, the functional roles of IRE1-bZIP17/bZIP28 in virus infection (indicated by “?”) have yet to be elucidated.

ATF6α and ATF6β are the members of type II ER trasmembrane proteins that possess bZIP transcription factor domains in their cytosolic regions (Haze et al., 1999). They are synthesized as inactive precursors, tethered to the ER membrane by an ER-targeting hydrophobic sequence (Figure 5). Unlike PERK and IRE1 which oligomerize upon ER stress, ATF6 translocates from the ER into the Golgi apparatus (Figure 5). Once translocated to the Golgi, it is proteolytically processed by Golgi-resident intramembrane proteases, first by site 1 protease (S1P) and then in an intramembrane region by site 2 protease (S2P) (Figure 5) (Hetz et al., 2011). This proteolytic processing releases its cytoplasmic DNA-binding domain, ATF6f (a fragment of ATF6), which operates as a transcriptional activator that upregulates many UPR genes related to protein folding (Figure 5) (Haze et al., 1999; Lee et al., 2002; Yamamoto et al., 2007).

As mentioned above, replication of HCV subgenomic replicons suppresses the IRE1-XBP1 pathway (Tardif et al., 2002, 2004). However, in cells infected by HCV replicons, subgenomic replication results in the activation of the ATF6 pathway, indicated by the presence of a 50 kDa protein, a cleavage product corresponding to the DNA-binding domain of ATF6 (Tardif et al., 2002, 2004). As a result, there is an increased transcriptional level of chaperones such as BiP. At present, it remains elusive which non-structural viral protein(s) are involved in induction of ATF6, since HVC subgenomic replicons only express the structural proteins. Other experiments suggest that the accumulation of unfolded MHC class I, which is attributed to a decline in protein glycosylation caused by HCV replication, might account for the activation of ATF6 (Tardif and Siddiqui, 2003). Additionally, acute infection with LCMV or expression of its glycoprotein precursor results in a selective induction of the ATF6-regulated pathway of the UPR, whereas pathways controlled by PERK and IRE1 are silent (Pasqual et al., 2011). It seems that a selective induction of the ATF6-regulated branch of the UPR is likely beneficial for virus replication and cell viability, whereas the induction of PERK and IRE1 may be detrimental for the invading virus and the host cell (Pasqual et al., 2011). Similarly, in Vero cell, ASFV induces the ATF6 signaling pathway, but not the PERK or IRE1 pathways, which might benefit the virus by assisting protein folding and preventing early apoptosis (Galindo et al., 2012).

A different pattern has been reported in cells infected with Hepatitis B virus (HBV) (Li et al., 2007). In Hep3B cells, expression of the multifunctional regulatory protein of HBV (HBx protein) alone is sufficient to activate both the ATF6 and IRE1-XBP1 pathways, and silencing HBx blocks their activation induced by the constitutive replication of HBV (Li et al., 2007). Therefore, HBx-mediated activation of these two pathways probably promotes HBV replication in liver cells. Similarly, both the IRE1 and ATF6 pathways are activated during Rotavirus infection (Trujillo-Alonso et al., 2011). Another scenario has also been found in human lung adenocarcinoma cells where a global UPR activation occurs upon DEN infection (Umareddy et al., 2007). Selective perturbation of the UPR pathways considerably alters DEN infectivity (Umareddy et al., 2007). Although the molecular mechanisms by which DEN infection activates ER stress remain to be elucidated, the three branches of the UPR signaling cascades might be hijacked by DEN to produce a condition beneficial to the viral infection.

Similar to animals, plants have signaling components that function in parallel to the IRE1-bZIP60 signaling cascade (Figure 5) (Urade, 2007; Vitale and Boston, 2008; Deng et al., 2011; Nagashima et al., 2011; Iwata and Koizumi, 2012). In *Arabidopsis*, bZIP transcription factors bZIP17 and bZIP28 are also synthesized as a precursor protein and anchored in the ER (Figure 5) (Iwata et al., 2008; Seo et al., 2008). In response to ER stress, bZIP17 and bZIP28 undergo proteolytic processing and translocation in a manner similar to the animal ATF6-S1P/S2P system (Figure 5) (Iwata and Koizumi, 2012). Upon translocated into the nucleus, bZIP17 and bZIP28 activate genes involved in the UPR and other signaling pathways such as brassinosteroid signaling transduction (Che et al., 2010). Although the proteolytic activation of bZIP17 and bZIP28 has been shown to be triggered by heat stress (Urade, 2007; Vitale and Boston, 2008; Deng et al., 2011; Nagashima et al., 2011; Iwata and Koizumi, 2012), no information is available at present about their roles in viral infection. Therefore, our understanding of the plant UPR pathway is very limited, and more efforts are needed to characterize the bZIP17/bZIP28 pathway and its roles in physiological and pathological settings.

### Crosstalk between three arms of the UPR

It is conceivable that IRE1, PERK, and ATF6 pathways communicate with each other extensively in many aspects, including activation, function, and feedback regulation. A seminal work discovering the crosstalk between these three arms comes from Hela cells, where XBP1 mRNA could be induced by ATF6 and spliced by IRE1 in response to ER stress (Yoshida et al., 2001). Moreover, transcriptional activation of XBP1 could be induced by the PERK signaling pathway as well, which might account for the broad effects of PERK during the UPR (Yoshida et al., 2001; Calfon et al., 2002). Besides PERK, IRE1 can also suppress protein translation via degrading mRNA (Hollien and Weissman, 2006; Hollien et al., 2009). In fact, a pro-apoptotic factor CHOP is regulated by both the ATF6 and PERK pathways (Schröder and Kaufman, 2005). While three arms of the UPR have their own specific functions in ER stress (Figures 3, 4, and 5), mutant analyses in *C. elegans* have revealed that the IRE1-XBP1 and the ATF6 arms of the UPR might activate a common set of genes involved in stress tolerance and worm development, indicating a functional redundancy between these two arms (Shen et al., 2005). Furthermore, all the three arms could induce ERAD (Schröder and Kaufman, 2005), representing a common cellular process resulting from the three UPR branches.

These crosstalks further add to the complexity of the UPR induced by abiotic and biotic cues such viral infection. For example, some viruses, such as HBV, Rotavirus, and DEN, usually activate two or even three pathways to promote reproduction (Li et al., 2007; Umareddy et al., 2007; Trujillo-Alonso et al., 2011). The expression of CMV Us11 or CMV infection inhibits the ATF6 pathway but activates the IRE1 pathway as an alternative mechanism to upregulate the expression of chaperones. Meanwhile, the transcriptional activation of the XBP1 target genes (e.g., those encoding protein degradation factors) regulated by the IRE1 pathway is inhibited, presumably in order to keep viral proteins in the ER from being degraded (Tirosh et al., 2005). In this case, it is puzzling how the virus activates the most favorable pathway for its replication and deactivates the molecular signaling pathway that is probably detrimental for its accumulation in the host cell.

So far, two UPR pathways have been identified in plants. Their crosstalk, however, does exist and appear diverse. The expression of *AtPDI* genes was found to decrease in the *AtbZIP60* mutant but not in the *AtIRE1-2* mutant, indicating that the additional UPR signaling complements AtbZIP60 in the activation of *AtPDI* gene expression during ER stress (Lu and Christopher, 2008). The structural similarity, especially in the putative transmembrane domain of the bZIP60, bZIP17, and bZIP28 proteins (Iwata and Koizumi, 2012), suggests that these two pathways might collaborate closely in sensing ER stress. Indeed, bZIP28 proteolytic activation and bZIP60 mRNA splicing could be induced concomitantly in response to heat stress (Gao et al., 2008; Deng et al., 2011). This assumption is also in agreement with another recent observation that bZIP28 is capable of forming a heterodimer with bZIP60 (Iwata et al., 2009; Liu and Howell, 2010), a direct crosstalk between these two pathways.

## Conclusion remarks

In higher eukaryotes, many critical biological processes are dependent on intercellular/intracellular communication, which requires relevant proteins timely and adequately expressed with high fidelity in folding. Therefore, the folding function of the ER and the signaling of the ER stress-induced UPR pathways have emerged as an important aspect of cell biology with broad implications to diverse physiological and pathological processes. Despite the recent advances made in understanding the UPR mechanisms implicated in abiotic and biotic stress such as viral infection, many critical questions still remain unanswered. The molecular and structural basis for recognition of the upstream signal by the ER stress sensors has only begun to be understood. Although several recognition models have been proposed mainly based on data using pharmacological chemicals and experimental stress conditions as the inducers of the UPR (Figure 2), we cannot empirically translate this knowledge into the case of viral infection. As discussed above, either virus replication or specific viral proteins (peptides) directly activate the UPR transducers, and different viruses may induce a specific UPR pathway(s). On the other hand, abiotic and biotic ER stress may also share some common UPR pathways that help host cells to defend against those adverse environmental stimuli. A good example is that virus infection can improve plant tolerance to abiotic stress (Xu et al., 2008). A key direction for future study in this field is to define how the ER stress is sensed and how those branched pathways are coordinated to function.

As a complex signal transduction network, the UPR protects the organisms against normal and unusual levels of ER stress by enhancing ER capacity, by reducing ER load, and by inducing programmed cell death. Different cell types may have different levels of sensitivity to ER stress. In response to specific viral infection and other stimuli, little is known about the regulation of UPR signaling in distinct cells, and how the kinetics and amplitude of signaling of each UPR branch is controlled. Our current knowledge about the roles of the downstream effectors of UPR transducers is also limited. For instance, it is unknown how the transcriptional activity of XBP1 is blocked in virus-infected cells (Figure 5). In plants, it is unclear whether the transcriptional activity of spliced bZIP60 is also a target by the invading virus, and whether there is an ERAD-like process responsible for removing spliced bZIP60 mRNA. As the plant IRE1 seems not only just to function through mediating bZIP60 mRNA splicing, its other downstream components remain to be characterized. A comprehensive study on these questions will certainly shed new lights in the UPR pathways, and assist in a better understanding of host–virus interactions and, in the long run, developing novel antiviral strategies.

### Conflict of interest statement

The authors declare that the research was conducted in the absence of any commercial or financial relationships that could be construed as a potential conflict of interest.

## Glossary

*Molecular chaperone*: A molecular chaperone is a protein that assists the folding/unfolding of other proteins. Some molecular chaperones reside in the lumen of the ER, such as BiP, also known as GRP78, a member of the Hsp70 family.
*Protein disulphide isomerase (PDI)*: A cellular enzyme in the lumen of the ER of eukaryotes or the periplasmic region of prokaryotes catalyzes the formation and breakage of disulphide bonds between cysteine residues within proteins, allowing proteins to quickly find the correct arrangement of disulfide bonds in their fully folded state.
*ER stress*: An organelles-initiated cell stress arises from mismatch between the load of unfolded or misfolded proteins in the lumen of the ER and the capacity of this cellular machinery.
*Unfolded protein response (UPR)*: A highly conserved physiological response is induced by accumulation of unfolded proteins in the lumen of the ER. In mammals, the UPR is mediated by three ER stress sensors including IRE1, PERK, and ATF6. In yeast, the UPR is controlled by only one signaling pathway mediated by IRE1. Thus far, two UPR pathways have been identified in plants, one mediated by IRE1-bZIP60 and the other by bZIP17/bZIP28.
*ER-assisted degradation (ERAD)*: ERAD is designated a cellular pathway, which translocates the unfolded proteins from the ER in a retrograde manner into the cytosol, where ER membrane associated ubiquitin ligases post-translationally modify the translocated proteins thereby targeting them for degradation, usually by the 26S proteasome.
*Programmed cell-death (PCD)*: The term PCD defines any form of cell death resulting from an orderly cascade, mediated by intracellular death programs, regardless of the triggers or the hallmarks it exhibits. PCD serves fundamental functions in both plants, and metazoans where called apoptosis.
*Major histocompatibility complex (MHC)*: An integral membrane protein complex has a characteristic groove as the binding site for the presentation of immunogenic peptides.
*Hemagglutinin-neuraminidase (HN)*: A single viral envelope glycoprotein has both receptor-cleaving and receptor-binding activity, which is in contrast to the protein found in influenza, where both hemagglutinin and neuraminidase activities reside in two separate glycoproteins.
*Eukaryotic translation initiation factor 2 (eIF2)*: The eIF2 complex is required in the translation initiation. It transfers Met-tRNA to the 40S submit of the ribosome to form the 43S pre-initiation complex in a GTP-dependent manner. eIF2 is a heterotrimer consisting of eIF2α, eIF2β, and eIF2γ. Phosphorylation of eIF2α by PERK inactivates eIF2α, resulting in inhibition of cap-dependent translation initiation.
*Unconventional processing*: Conventional splicing is catalyzed by the spliceosome, which is composed of multiple proteins and small nuclear RNAs, and the cleavage reaction proceeds sequentially. The nucleotide sequence at the exon–intron border complies with Chambon's rule (GU-AG rule). In contrast, unconventional splicing is catalyzed by IRE1 and tRNA ligase, which is independent of the spliceosome, and the order of cleavage of the exon–intron junctions is not predetermined. A pair of characteristic stem–loop structures exists at the cleavage sites, which is recognized by IRE1, instead of a consensus sequence such as GU-AG.
